# Evaluating potential sources of invasive wild pigs in Ontario

**DOI:** 10.1002/ece3.8160

**Published:** 2021-09-27

**Authors:** Erin L. Koen, Erica J. Newton, E. Hance Ellington

**Affiliations:** ^1^ Wildlife Research and Monitoring Section Ontario Ministry of Northern Development Mines, Natural Resources, and Forestry Peterborough ON Canada; ^2^ Department of Wildlife Ecology and Conservation Range Cattle Research and Education Center University of Florida Ona Florida USA

**Keywords:** Canada, feral swine, invasion, Ontario, *Sus scrofa*, wild boar farm

## Abstract

Invasive wild pigs (*Sus scrofa*) are considered one of the most damaging species globally, and once they become established in an area, they are notoriously difficult to eliminate. As such, identifying the potential pathways of invasion, especially in places with emerging populations, is critical for preventing new or continued invasion. Wild pigs have been reported in Ontario, Canada, in recent years. We tested four nonexclusive hypotheses about the source of wild pigs in Ontario: (a) escapees from captive sources within Ontario; (b) invasion from neighboring jurisdictions; (c) existing wild populations within Ontario; and (d) translocation and illegal release. We found that sightings of Eurasian wild boar were closer to premises with wild boar than were random locations; wild boar sightings were an average of 16.3 km (*SD* = 25.4 km, min = 0.2 km, *n* = 20) from premises with wild boar. We also found that sightings of domestic pigs were closer to domestic pig farms than expected. Sightings of wild pigs in groups of more than four animals were rare. Our results suggest that wild pigs observed in Ontario are recent escapes from captivity, recognizing that there may be established groups of wild pigs that we have not yet detected. While not common, we also received reports indicating that in the past, wild pigs have been translocated and illegally released. Other North American jurisdictions that have been successful at eliminating wild pigs have removed existing populations and changed regulations to limit future invasion, such as prohibiting possession and transport of wild boar and prohibiting hunting of wild pigs.

## Graphical abstract

Invasive wild pigs are considered one of the most damaging species globally and are notoriously difficult to eliminate. Identifying the potential pathways of invasion, especially in places with emerging populations, is critical for preventing new or continued invasion. Our results suggest that wild pigs observed in Ontario, Canada, are recent escapes from captivity.

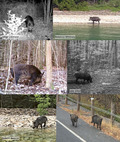

## 1 INTRODUCTION

Once an invasive species has been introduced and becomes established in a region, eradicating that species becomes increasingly expensive and, depending on the species, may be financially and logistically unfeasible (Parkes & Panetta, 2009; Vitousek et al., 1996). Successful control strategies for invasive species involve a combination of preventing the introduction of the invader, detecting early invaders, and rapidly removing those early invaders (Mehta et al., 2007). Preventing the introduction and subsequent establishment of an invasive species, while challenging (Mehta et al., 2007), is the most efficient method to prevent widespread invasion (Leung et al., 2002; Reaser et al., 2020). Identifying the potential pathways of invasion is thus a critical step for preventing new or continued invasions.

Wild pigs (*Sus scrofa*) have successfully invaded every nonpolar continent worldwide (Barrios‐Garcia & Ballari, 2012; Lewis et al., 2017; Mayer & Brisbin, 2008). They are considered one of the most damaging invasive species globally (Lowe et al., 2000), having a massive negative effect on biodiversity worldwide (Bellard et al., 2016; Doherty et al., 2016). The distribution of invasive wild pigs has been steadily increasing in both the United States (Corn & Jordan, 2017; McClure et al., 2015; Snow et al., 2017) and parts of Canada (Aschim & Brook, 2019). We define a wild pig as any member of *S. scrofa* outside of a fence, including pigs phenotypically resembling Eurasian wild boar, domestic pig breeds including pot‐bellied pigs, and/or hybrids. Most wild pigs in the United States are of mixed ancestry (western heritage breeds of domestic pig and European populations of wild boar), suggesting that hybrids might have increased invasive potential (Smyser et al., 2020). All pigs, regardless of ancestry, can interbreed and have the potential to become feral (e.g., Caudell et al., 2013).

In many parts of North America, invasion by wild pigs into new areas is due to range expansion from existing populations (Snow et al., 2017), escapes from farms and high‐fence shooting operations (e.g., Jackling et al., 2016), and illegal release of wild pigs by humans to establish hunting opportunities in new areas (McCann et al., 2018; Tabak et al., 2017; Waithman et al., 1999). In Saskatchewan, Canada, among the strongest predictors of wild pig distribution is proximity to Eurasian wild boar farms (Michel et al., 2017).

Wild pigs have been reported in Ontario, Canada, in recent years (Aschim & Brook, 2019; Koen & Newton, 2021). We examined four nonexclusive hypotheses of the source of wild pigs in Ontario (see also Section 2.4): (a) escapees from captive sources within Ontario; (b) invasion from neighboring jurisdictions; (c) existing wild populations within Ontario; and (d) translocation and illegal release. Farming of wild boar for meat currently takes place in Ontario, although fenced hunting facilities have been banned since 2005. Ontario also shares borders with two Canadian provinces and three US states, all of which currently have wild pigs or have had them in the past. We tested each hypothesis on the three different types of wild pigs sighted in the province—wild boar, domestic pigs, and pigs of unknown ancestry, using a database of wild pig sightings reported by community members. Knowledge of the probable source of wild pigs in Ontario will help to guide future policies and management plans aimed at preventing the establishment of wild pigs.

## 2 METHODS

### 2.1 Study area

**FIGURE 1:**
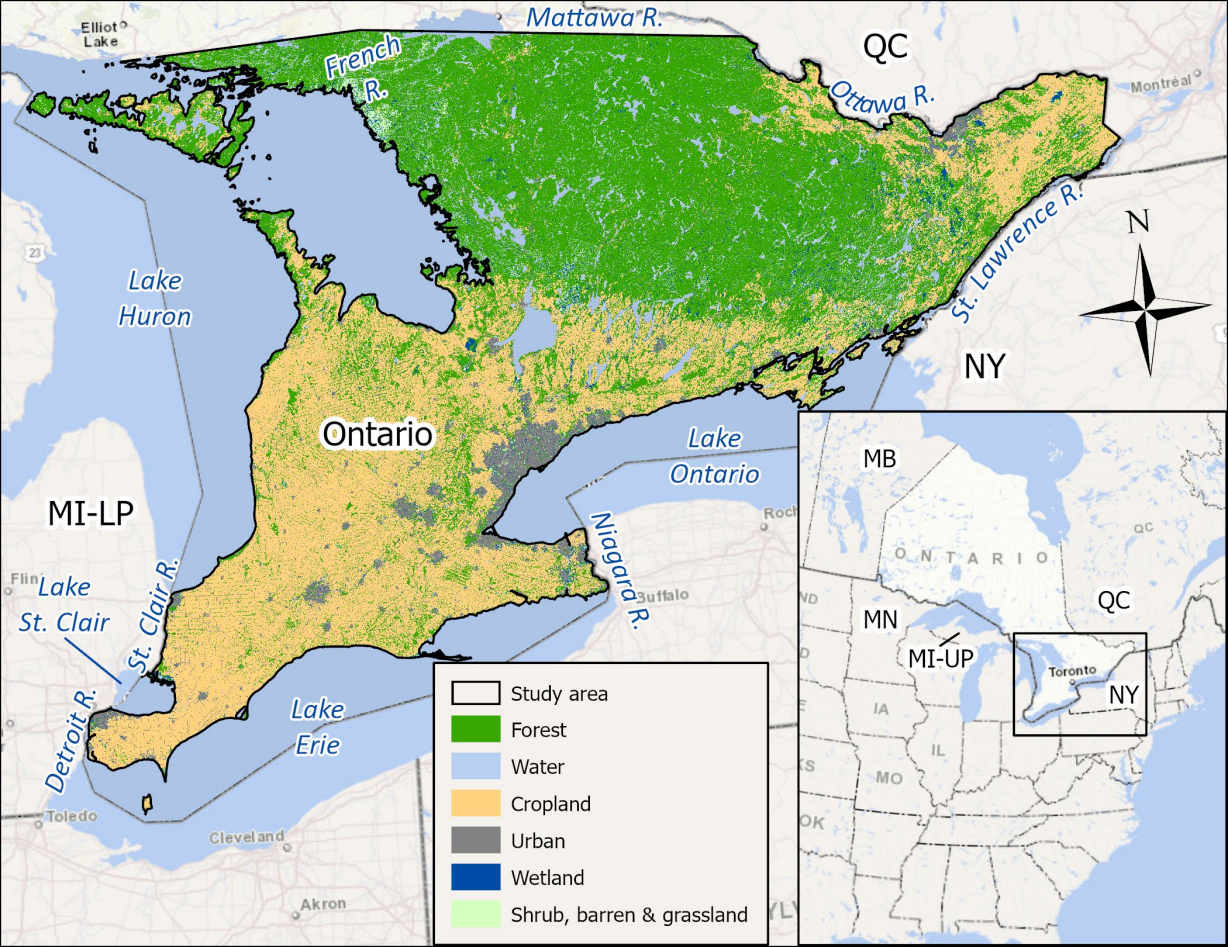
Predominant land cover (Natural Resources Canada, 2019), lakes, and major rivers in the study area in Ontario, Canada

We focused our study in Ontario, Canada, south of the French and Mattawa Rivers (~136,000 km^2^; Figure 1) where 96% of the sightings in our database occurred. There is a lower human population density in northern Ontario; thus, there was a lower detection probability north of our study area. The study area was comprised of forest (35%), water (30%), cropland (25%), urban (6%), wetland (2%), and shrubland, barren, and grassland (2%) (Figure 1; Natural Resources Canada, 2019).

### 2.2 Approach

Our aim was to identify the most likely source of observed wild pigs in Ontario by comparing support for four nonexclusive hypotheses using our database of reported wild pig sightings. We used a weight of evidence approach, as defined by Hardy et al. (2017), whereby we integrated several lines of evidence to weigh relative support for each hypothesis. As such, we considered rankings from Akaike information criterion (AICc) corrected for small sample size (Burnham & Anderson, 2002), effect size, information obtained from trail camera images, and knowledge from informal conversations with residents and local officials. We expected that the different types of data would offer support for some or all of the hypotheses. These hypotheses are not mutually exclusive, as it is possible that wild pigs in Ontario are coming from several sources.

### 2.3 Wild pig sightings

In October 2018, we (Ontario Ministry of Northern Development, Mines, Natural Resources, and Forestry (NDMNRF) staff) set up a provincial reporting and monitoring program for invasive wild pigs in Ontario, which included a dedicated email address to receive sightings from the public. We used outreach to solicit sightings from community members, including an informational website, print and online news, radio, social media, and fact sheets, with directions for the public on where to report sightings of any wild pig observed in the province (Koen & Newton, 2021). Our outreach efforts targeted both provincial and local audiences.

We collected 264 wild pig sightings that were observed on the landscape between July 2009 and the end of March 2021 in Ontario from several sources: (a) sightings reported to the NDMNRF by the public (71.6%); (b) sightings obtained from iNaturalist (https://www.inaturalist.org/projects/ontario‐wild‐pig‐reporting; 4.9%); (c) sightings gleaned opportunistically from online blogs or detailed stories in the media (4.9%); (d) an independent wild pig reporting website (www.wildboarcanada.ca; 13.3%); and (e) sightings reported to another agency (5.3%). We verified 127 of these sightings with submitted photographs or with site visits by NDMNRF staff. We did not include reports if we were not confident it was a pig (e.g., a sighting of a track that looked similar to the more locally abundant white‐tailed deer (*Odocoileus virginianus*)) or when we suspected duplicate reports of the same individual wild pig(s) submitted independently by different observers (e.g., photographs of a pot‐bellied pig with unique markings, or a group of three domestic pigs, taken in the same area within the same week).

**FIGURE A1:**
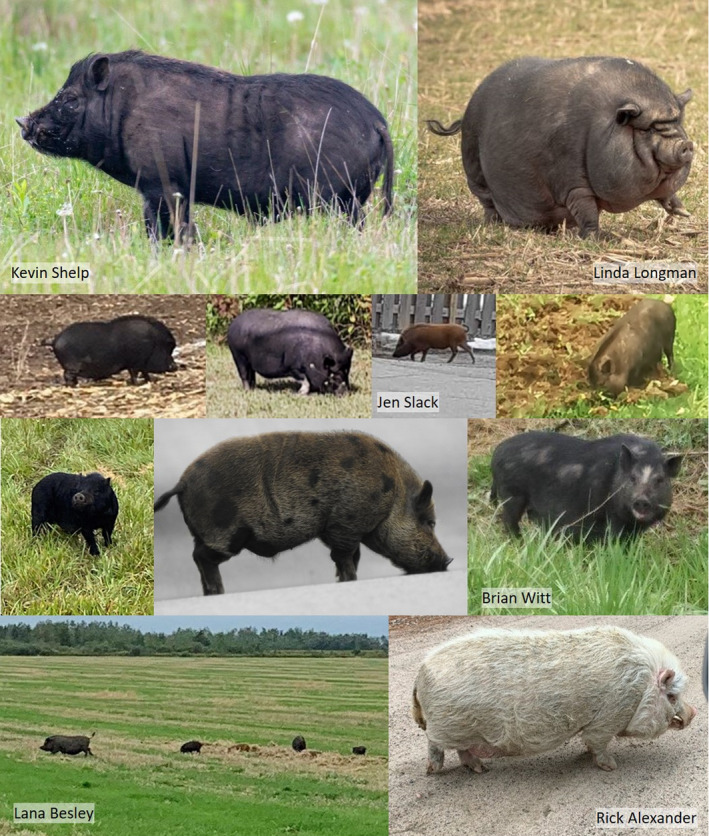
Photographs of wild pigs (*Sus scrofa*) that phenotypically resemble or were verified to be pot‐bellied pigs in Ontario, submitted by the public to the Ontario Ministry of Northern Development, Mines, Natural Resources, and Forestry. Permission to use these photographs has been provided from the photographers (names listed below each photograph, otherwise anonymous)

**FIGURE A2:**
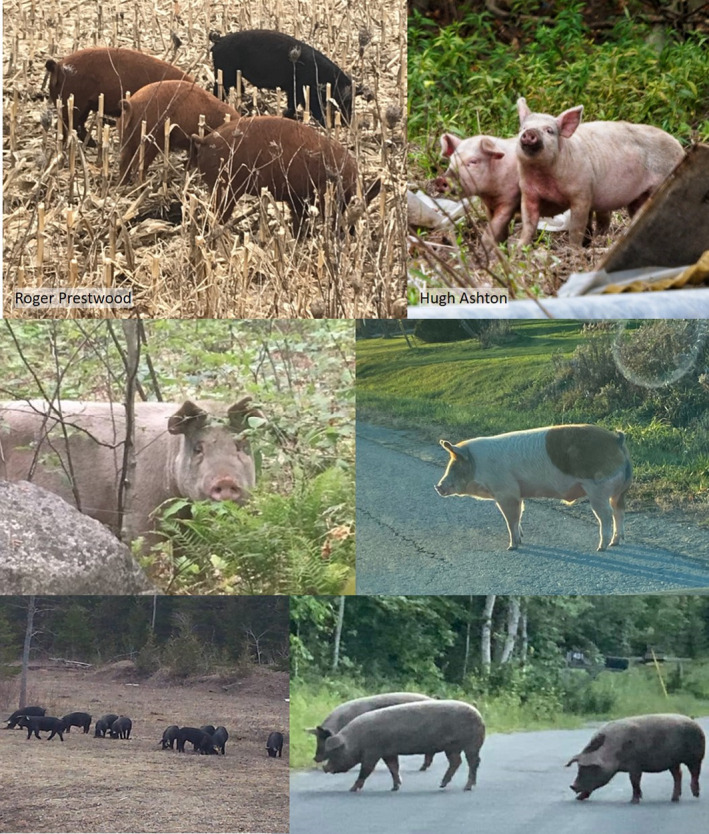
Photographs of wild pigs (*Sus scrofa*) that phenotypically resemble or were verified to be domestic pigs in Ontario, submitted by the public to the Ontario Ministry of Northern Development, Mines, Natural Resources, and Forestry. Permission to use these photographs has been provided from the photographers (names listed below each photograph, otherwise anonymous)

**FIGURE A3:**
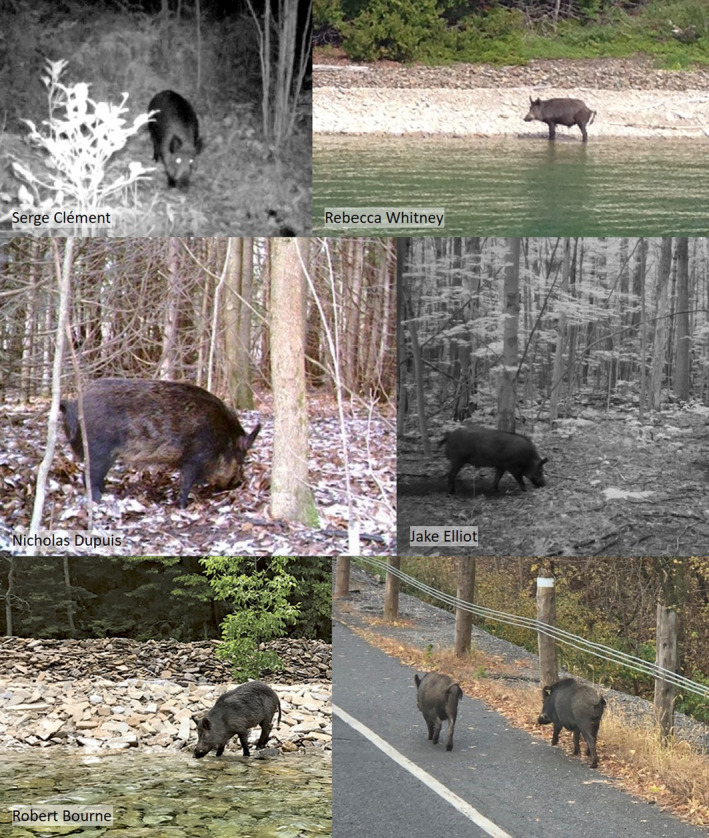
Photographs of wild pigs (*Sus scrofa*) that phenotypically resemble or were verified to be Eurasian wild boar in Ontario, submitted by the public to the Ontario Ministry of Northern Development, Mines, Natural Resources, and Forestry. Permission to use these photographs has been provided from the photographers (names listed below each photograph, otherwise anonymous)

When sightings were accompanied by photographs, we classified those sightings, when possible, as Eurasian wild boar or domestic breeds (including pot‐bellied pigs) based on phenotype. For the remainder, we did not have a photograph and thus we classified them as an “unknown” type. Most wild pigs in the United States are hybrids of Eurasian wild boar and western heritage breeds of domestic pig (e.g., Tamworth, Ossabaw, mulefoot; Smyser et al., 2020). In Ontario, however, we feel confident that the majority of submitted photographs can be qualitatively classified by pig type (e.g., Figures A1, A2, A3), although we acknowledge that our classifications would be strengthened with genetic data.

### 2.4 Hypotheses

#### 2.4.1 Escapes from captive populations

If the source of observed wild pigs is escapees from captive populations within Ontario, we expected that wild pig sightings would be closer to premises that have pigs compared with the distance between random locations and these premises. Thus, we generated several univariate logistic regression models comparing: (a) the distance between sightings of wild boar and premises with wild boar; (b) the distance between sightings of domestic pigs and domestic pig farms; and (c) the distance between pigs of unknown ancestry and (a) premises with wild boar; and (b) domestic pig farms. We used univariate models because we did not have enough sightings of wild boar to support the use of multivariate models. We also expected to find, through site investigations, that many reported wild pigs would be owned and recently escaped, rather than free‐living or feral.

##### Premises with captive wild boar

We mapped the locations of premises with wild boar in Ontario that we collected opportunistically using four strategies: (a) we scanned the Internet in July 2019, February 2020, August 2020, December 2020, and March 2021 for premises that advertised the presence of live wild boar (22.8%); (b) we scanned Kijiji (a Canadian online platform for local buying and selling) once per week in 2019, 2020, and January–March 2021 for advertisements of people selling live wild boar or wild boar meat (40.4%); (c) we identified premises with wild boar from word‐of‐mouth conversations with landowners and hunters (28.1); and (d) we obtained locations of premises with domestic wild boar from the Ontario Ministry of Agriculture, Food, and Rural Affairs (8.8%), for a total of 57 active or inactive premises. For half of these premises, we estimated that the accuracy of the location of that premise in our dataset was within 2 km. For the other half, we do not know the exact location of the premise and our estimated location could be within 10 km of the actual premise. Three additional premises were in the province of Quebec <5 km from the Ontario border, but we did not include these in our analysis because we tested a separate hypothesis that wild pigs in Ontario were invading from other jurisdictions. Of the 57 premises, 51 (89.5%) were farms advertising the sale of live wild boar or wild boar meat products. The remaining premises were zoos or currently inactive captive wild boar hunting operations. These now defunct operations could have been the source of escaped wild boar observed more recently and reported to us by the public. We also included one island in our study area with a reported wild boar population (see results for more information) that might be a source of wild boar on the mainland (2 km away) if they escaped the island. We excluded reports of wild pigs on this island from all distance models, as we considered the location, for our purposes here, as a potential “source.”

We acknowledge that our dataset of potential sources of wild boar is incomplete because there are likely other premises with wild boar that we do not yet know about. We also note that there is variability among premises that we did not account for with respect to risk (e.g., number of swine and fence strength and security) because these data were unavailable. We did not include Census of Agriculture data in our analyses, which provides a count of many of the active wild boar farms at the scale of census subdivision up until census year 2011 (Michel et al., 2017; Statistics Canada, 2008); for this study, we required point locations of premises with wild boar at a finer resolution than census subdivision.

##### Domestic pig farms

We obtained the locations of 6,050 premises farming domestic pigs in Ontario (unpublished data, the Ontario Ministry of Agriculture, Food, and Rural Affairs). We note that smaller hobby farms may be more likely to raise domestic pigs (including pot‐bellied pigs) outdoors, increasing escape risk, but not all small farms are registered or present in this dataset, and we do not know locations of most pot‐bellied pig owners.

#### 2.4.2 Invasion from neighboring jurisdictions

If the source of observed wild pigs in Ontario is expansion from wild pig populations in neighboring jurisdictions, we expected that wild pig sightings would be closer to Ontario's borders than are random locations. Thus, we compared the distance between wild pig sightings (separately for wild boar, domestic pigs, and pigs of unknown ancestry) and the nearest point along each of Ontario's shared borders with Quebec (QC), Canada; New York (NY), USA; and the lower peninsula of Michigan (MI‐LP), USA (Figure 1). We do not have data on the locations of potential wild pig sources in these areas (except for three wild boar farms in Quebec close to Ontario's border noted above); therefore, this is an overestimate because it assumes that each border has wild pigs on the other side. This is unlikely to be true, but overestimating this source gives us confidence that wild pigs are not likely invading Ontario from neighboring jurisdictions if we cannot detect even this signal. The majority of borders between Ontario and neighboring jurisdictions are along waterways: the Ottawa River separates much of Ontario from QC, the St. Lawrence and Niagara Rivers separate Ontario from NY, and the St. Claire and Detroit Rivers separate Ontario from MI‐LP (Figure 1). We considered only sections of the borders separated by a waterway of ≤5 km; this cutoff retained much of the border between the jurisdictions but excluded large lakes and rivers >5 km wide as possible pathways for wild pigs to enter Ontario (Figure 1). Wild pigs are capable of both swimming (Hammell et al., 1975; Rawlinson et al., 1992) and crossing the narrower sections of these waterways in colder winters by walking across ice bridges.

We did not include borders with Manitoba, Canada (MB); Minnesota, USA (MN); and the upper peninsula of Michigan (MI‐UP) (Figure 1) because they did not border our study area and because we have a lower probability of detection at the northern edge of our study area.

#### 2.4.3 Existing populations

Wild pigs in established populations tend to live in social groups composed of single or multiple family groups (Mayer, 2009). We expected that if wild pig sightings were associated with established wild pig populations within the province, that sightings of larger family or social groups of wild pigs with young would be detected, as observed in established populations of wild pigs in other parts of Canada (Koen et al., 2018; Stolle et al., 2015) and the United States (Mayer, 2009). For example, 45% of collared individuals in Saskatchewan, Canada, where wild pig populations are self‐sustaining and spreading, were in groups made up of more than four individuals (Koen et al., 2018), and group size in established wild pig populations in the United States tend to range from 3 to 9 individuals (Mayer, 2009). We present the number of wild pigs observed in each sighting to investigate whether wild pigs were more often seen alone or in small groups (showing support for the recent escapes hypothesis) or commonly observed in larger social groups (showing support for the established population hypothesis). We acknowledge, however, that given our sampling method (reports from the public), we are more likely to detect pigs that are active during the day and those that behave less secretively. As such, observing mostly individual or small groups of pigs does not necessarily mean that large sounders of wild pigs do not exist—they may simply be undetected at this time. We investigated concerning sightings using our own baited trail cameras, searching for sign, and we canvassed the area speaking with locals—these investigations refined our estimates of group size.

#### 2.4.4 Translocation

If wild pigs have been translocated and illegally released to create new hunting opportunities, as has been documented in the United States, we expected to receive reports of Eurasian wild boar in groups with more than one individual. We would anticipate that >1 individual would be released in an area, and we would expect those individuals to be of full or partial Eurasian wild boar ancestry because this is the phenotype typically stocked in fenced hunting enclosures (as opposed to pot‐bellied pigs or domestic pigs). We also expected to receive information gleaned from conversations with residents about this practice occurring.

### 2.5 Statistical analyses

To evaluate our nonexclusive hypotheses, we used a weight of evidence approach, where we considered several lines of evidence to compare relative support for each hypothesis. We used binomial univariate logistic regression models to assess support for two of our hypotheses (escape from captivity and invasion from neighboring jurisdictions) using program R (R Core Team, 2021). We compared the locations of sightings, separated by type (wild boar *n* = 20, domestic pig *n* = 108, and unknown *n* = 135) to 250 random locations within our study area. To represent the escape from captivity hypothesis, we estimated the distance to sources of captive pigs (wild boar or domestic pig), and to represent the invasion from neighboring jurisdiction hypothesis, we estimated the distance to neighboring jurisdictions (New York, Quebec, and the lower peninsula of Michigan). We then compared relative support for univariate models within each sighting type using AICc with package AICcmodavg (Mazerolle, 2016) in R. We also included null models in the analysis. For variables that were important in explaining variation in the location of pig sightings, we used Cohen's *d* (Cohen, 1988; Lakens, 2013) to calculate the size of the effect (relative to the mean for random locations), where *d* = 0.2 is considered a small effect, *d* = 0.5 is considered a medium effect, and *d* = 0.8 is considered a large effect. We repeated this analysis with 2,000 random locations, and our conclusions were similar (Tables A1, A2 and A1, A2), suggesting that our analysis was not sensitive to the number of random locations used. We report only the findings based on comparison to 250 random locations.

We tested for correlation among variables using Pearson's r in program R. Distance to a premise with wild boar was moderately correlated with the distance to a pig farm (*r* = 0.58) and to the NY border (*r* = 0.54). The distance to the MI‐LP border was negatively correlated with the distance to the QC border (*r* = −0.95). We retained all variables as we did not run multivariate models. We used the packages dplyr (Wickham et al., 2020), ggplot2 (Wickham, 2016), sf (Pebesma, 2018), sp (Bivand et al., 2013), raster (Hijmans, 2020), and rgdal (Bivand et al., 2019) in program R for data manipulation, spatial analyses, and figure creation.

### 2.6 Site investigations

We followed up on all reported sightings via email or phone to gather as much information as possible about the circumstances surrounding the report, when contact information was available. In some cases, we followed up again months later to find out if additional information had become available. We investigated some sightings with a site visit, prioritizing reports of pigs that phenotypically resembled Eurasian wild boar, multiple pigs or pigs with young, pigs that had been present on the landscape for longer periods of time (several months or more), and/or reports of pigs causing damage.

Between January 2020 and March 2021 inclusive, we investigated 33 locations in Ontario where one or more wild pig reports had been received. At these locations, our investigations involved conversations with residents and local officials and in some cases, we set up trail cameras baited with corn. In total, we had 473 conversations, left notes in an additional 925 residences (with information on how to contact us to discuss past or future sightings), and set up 64 baited trail cameras. Our effort varied across sites depending on the nature of the report.

## 3 RESULTS

### 3.1 Escapes from captivity and invasion from neighboring jurisdictions

**FIGURE 2:**
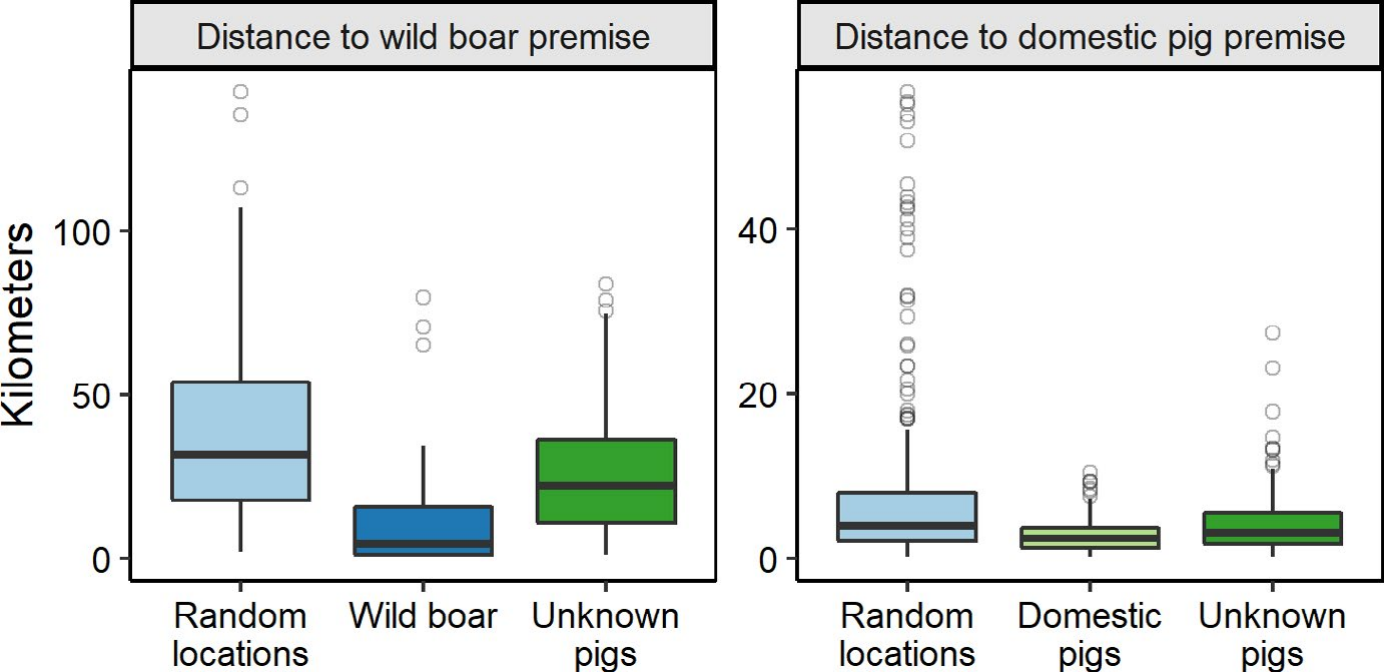
Distance of each location (random locations, wild boar, domestic pigs, and unknown pigs) to premises with domestic pigs or wild boar farms in Ontario, Canada. Sightings were reported from October 2018 to March 2021, but wild pigs were observed on the landscape as early as 2009. Boxplots show the median, the 25th and 75th percentiles (hinges), the smallest and largest values no further than 1.5 times the interquartile range from the hinge (whiskers), and outliers (points)

The top univariate model predicting the location of wild boar sightings was distance to a premise with wild boar (Table 1); wild boar sightings tended to be closer to premises with wild boar than were random locations (Figure 2, Table 2). Wild boar sightings were an average of 16.3 km (*SD* = 25.4, median = 4.2 km, range = 0.2–79.7 km, *n* = 20) from the nearest premise with wild boar; the size of the effect relative to random locations (mean = 37.5 km, *SD* = 25.3, *n* = 250) was large (*d* = 0.84; Figure 2).

**TABLE 1:** Univariate models predicting the location of wild pig (*Sus scrofa*) sightings in Ontario, Canada observed between July 2009 and March 2021, grouped by “type” of wild pig, compared with 250 random locations

Model^a^	*K*	Wild boar [*n* = 20]			Domestic pig [*n* = 108]			Unknown pig [*n* = 135]		
		AICc	ΔAICc	*w_i_*	AICc	ΔAICc	*w_i_*	AICc	ΔAICc	*w_i_*
Distance to premise with wild boar	2	**129.1**	**0**	**1.00**				**483.1**	**0**	**0.60**
Distance to domestic pig farm	2				**401.6**	**0**	**1.00**	**484.0**	**0.85**	**0.39**
Distance to border (NY)	2	143.4	14.25	0	438.2	36.60	0	493.8	10.62	0
Distance to border (MI‐LP)	2	145.0	15.86	0	425.4	23.73	0	497.7	14.59	0
Distance to border (QC)	2	146.1	16.98	0	418.0	16.34	0	495.0	11.86	0
Null model	1	144.6	15.48	0	440.4	38.77	0	500.9	17.71	0

Note

Univariate models within 2 ΔAICc are in bold font.

^a^ Our study area in Ontario shares borders with Quebec, Canada (QC); New York, USA (NY); and the lower peninsula of Michigan, USA (MI‐LP). Ontario also shares borders with Manitoba, Canada, Minnesota, USA, and the upper peninsula of Michigan, USA, but these regions were not included in our study area.

The top univariate model predicting the location of domestic pig sightings was distance to a domestic pig farm (Table 1). Domestic pig sightings were, on average, 3.0 km (*SD* = 2.3 km, median = 2.4 km, range = 0.2–10.4 km, *n* = 108) from a domestic pig farm, with a medium effect size relative to random locations (*d* = 0.55; Figure 2).

The top model predicting the location of unknown types of wild pigs was distance to a wild boar farm (Table 1). Sightings of pigs with unknown ancestry were an average of 26.5 km (*SD* = 19.8, median = 22.1 km, range = 1.0–83.8 km, *n* = 135; Figure 2, Table 2) from the nearest premise with wild boar, with a medium effect size relative to random locations (*d* = 0.47; Figure 2). There was also support for the model suggesting that sightings of pigs with unknown ancestry tended to be closer to domestic pig farms (Table 1, Table 2, Figure 2); they were an average of 4.3 km (*SD* = 4.2, median = 3.1 km, range = 0.1–27.5 km, *n* = 135) from the nearest domestic pig farm, with a medium effect size (*d* = 0.42; Figure 2).

**TABLE 2:** Coefficients [*SE*] from univariate models predicting the location of wild pig (*Sus scrofa*) sightings in Ontario, Canada, observed between July 2009 and March 2021, grouped by “type” of wild pig, compared with 250 random locations

Variable^a^	Wild boar [*n* = 20]	Domestic pig [*n* = 108]	Unknown pig [*n* = 135]
Distance to premise with wild boar	**−0.056 [0.017]**		**−0.022 [0.005]**
Distance to domestic pig farm		**−0.188 [0.044]**	**−0.069 [0.020]**
Distance to border (MI‐LP)	**0.002 [0.001]**	**−0.003 [0.001]**	**−0.002 [0.001]**
Distance to border (NY)	**−0.005 [0.003]**	**−0.003 [0.001]**	**−0.004 [0.001]**
Distance to border (QC)	−0.001 [0.002]	**0.004 [0.001]**	**0.002 [0.001]**

Note

Coefficients with variance that does not overlap zero are in bold font. We measured distances in kilometers.

^a^ Our study area in Ontario shares borders with Quebec, Canada (QC); New York, USA (NY); and the lower peninsula of Michigan, USA (MI‐LP). Ontario also shares borders with Manitoba, Canada; Minnesota, USA; and the upper peninsula of Michigan, but these areas were not included in our study area.

For all three types of pigs, there was little support for models relating pig locations to borders of neighboring jurisdictions (Table 1).

Through follow‐up of the wild pig sightings included in our analysis, we learned that many of the reports were of pigs that had escaped captivity or were owned, including pet pot‐bellied pigs, farmed Eurasian wild boar, or escaped domestic pigs. In one example, we received a report of two dead wild pigs, and through investigation learned that they were someone's pet pot‐bellied pigs that had been shot by hunters. In another example, we received a report of three dead wild pigs, and through follow‐up we learned that these Eurasian wild boar carcasses were illegally dumped by the owner 15 km from their farm. In total, 13.9% of sightings were of owned pigs. We did not investigate 62.5% of reports, however, and we did not prioritize follow‐up of sightings of domestic pigs. Therefore, 13.9% is likely an underestimate of the number of reports that were of owned pigs. While we did encounter some free‐roaming pigs that did not appear to have an owner, these were comparatively rare and isolated in both space and time.

### 3.2 Existing populations

**FIGURE 3:**
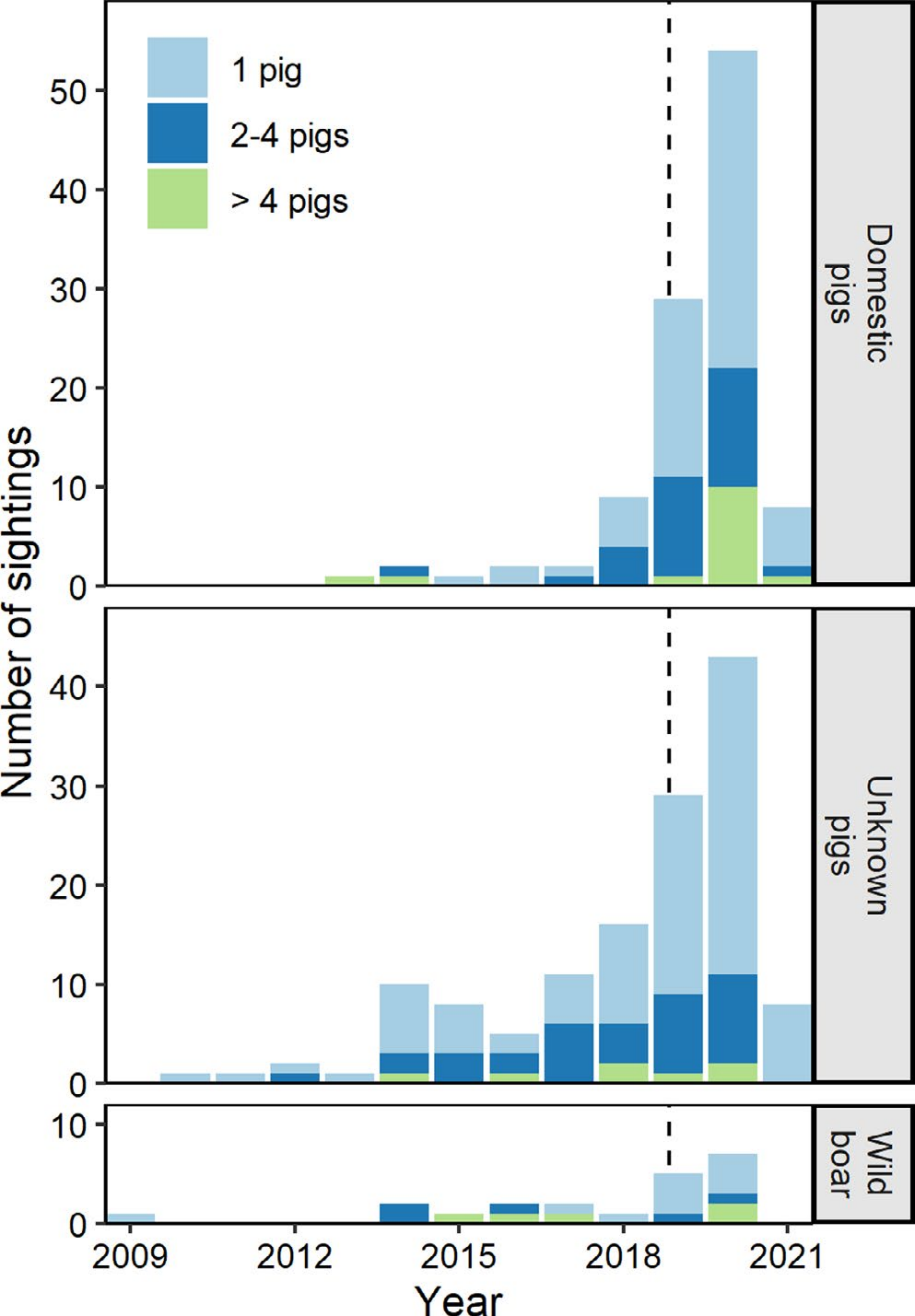
Number of wild pigs (domestic pigs, unknown pigs, and wild boar) observed in each sighting in Ontario, Canada, including pigs reported dead (17.0% of sightings included >1 dead pig). We collected sightings from October 2018 (dashed line) to March 2021, but these reported pigs were observed on the landscape as early as 2009; the apparent increase in pig sightings is likely due to the commencement of our monitoring program in 2018 and an increase in effort to solicit sightings (Koen & Newton, 2021)

We predicted that if wild pig sightings in Ontario were associated with established wild pig populations within the province, that sightings of larger family groups would be noted. Wild pigs in Ontario were more commonly found alone (64.0% of all sightings), and only 9.8% of all sightings were of groups numbering more than four pigs (Figure 3); however, some of these were reported dead. Domestic pigs were also most often reported alone (60.2% of all domestic pig sightings); 13% were of groups numbering more than four. In Ontario, smallholder farmers may use domestic pigs for crop residue grazing; in some cases, these pigs (observed in groups of 20–50 individuals) were reported to us by concerned citizens but most were later confirmed to have returned to their enclosures. Alternatively, wild boar were most often reported dead (57.1% of all wild boar sightings), and there were only two sightings since 2018 of more than four verified wild boar observed alive together (one group were farm escapes and were later recaptured; the other group was reported on an island). Wild pigs of unknown ancestry were most commonly seen alone (68.9%) and very rarely in groups with more than four animals (5.2%). Further, 11.7% of our sightings were of the same pig(s) reported multiple times by different community members, suggesting that in some parts of the province, we are likely to detect wild pigs if they are present. We expect that if a large group or population of wild pigs was present in a place with adequate detection probability, reports of these groups would occur clustered in both time and space. Except for several groups reported (and removed) between 2014 and 2018, and reports from one island, this has not been the case in recent years in Ontario.

### 3.3 Translocation

Through conversations with local wildlife officials, we learned of one location in Ontario where Eurasian wild boar had been translocated several decades ago and released onto a privately owned island to create an exclusive hunting opportunity. We have received verified reports over the past decade of single animals and family groups of wild boar on the island. We have also received sightings of wild boar and unknown types of pigs on the mainland (which is 2 km from the island) over the past 10 years, but there is no indication at this time that individuals on the island have seeded new populations on the mainland. We also received three separate reports of occasions over the past two decades where captive wild boar were illegally released elsewhere in the province, either for hunting or after the ban on fenced hunting operations.

### 3.4 Site investigations

For sightings of particular concern, we set 64 baited trail cameras at 51 sites within 12 locations, for a total of 2,432 active camera trap nights. We detected wild pigs at only four of these cameras (two locations), and all of these pigs were later humanely euthanized or recaptured by our staff or by the owners of the pigs. In total, 29.2% of the 264 reports were resolved through remote or on‐site investigation (e.g., we learned that pigs were contained, removed, killed, died, or otherwise no longer classed as “wild” pigs).

## 4 DISCUSSION

Our results suggest that the main source of observed wild pigs in Ontario is escapes from captive populations. We did not find evidence that wild pigs are invading Ontario from neighboring jurisdictions at this time. Likewise, we did not find evidence of existing wild pig populations in mainland Ontario. We found some evidence that wild pigs have been translocated and illegally released within Ontario to create hunting opportunities in the past, although we have not seen evidence that these events have seeded new populations.

We found support for the hypothesis that observed wild pigs in Ontario have originated from captive populations, presumably as escapes from captivity. Wild boar sightings tended to be closer to premises with wild boar than were random locations. Similarly, domestic pig sightings tended to be closer to domestic pig farms than were random locations. Interestingly, sightings of pigs with unknown ancestry (unknown because we did not have a photograph of the sighting) tended to be closer to premises with wild boars, suggesting that some of the unknown wild pigs might have been of wild boar ancestry; however, there was also strong support for unknown pigs being closer than random to domestic pig farms. This finding is further supported by our follow‐up investigations, where we learned by speaking with residents and local farmers that many of the pig sightings we received were, in fact, recent escapes from captivity. We expect that our estimate of the proportion of sightings that are recent escapes is a significant underestimate, as the sightings that we prioritized for follow‐up were often pigs that we were concerned might be feral; we were less likely to follow up on sightings that we suspected to be recent escapes.

We did not find support for the hypothesis that the source of observed wild pigs in Ontario is invasion from neighboring jurisdictions. The shape of the Ontario landmass, with most provincial borders bounded by lakes and rivers (Figure 1), means that Ontario is somewhat protected from invasion at its southern and eastern borders (e.g., Rees et al., 2009). Furthermore, officials in New York state, which borders Ontario to the south and the southeast, have successfully eliminated wild pigs from the state as of 2014 (Jackling et al., 2016), meaning that invasion from New York is unlikely because there are currently no known wild pig populations there. Likewise, wild pigs in Michigan occur in localized areas and at relatively low population densities (Gray et al., 2020). We did not assess support for the hypothesis that Manitoba (which has localized populations of wild pigs; Aschim & Brook, 2019) or Minnesota (which does not have any known wild pig populations; Brook & Glow, 2020) are sources of wild pigs in Ontario; there is a lower human population density in northern and northwestern Ontario to report sightings, so our surveillance effort was lower in those areas. While there are reports of wild pigs in Quebec (Aschim & Brook, 2019), we did not find support for the hypothesis that wild pigs in Ontario are invading from Quebec. We note that the Ottawa River separates much of Ontario from Quebec and might impede crossing. We did receive a report, however, of a wild pig swimming between mainland Ontario and an island in the Ottawa River. We know of three premises with wild boar in Quebec within 5 km of the Ontario border and that a wild boar sighting in Ontario was 4.6 km from one of these premises in Quebec. As such, although we did not find support for the model testing invasion from Quebec, it is possible that some wild boar escapees from Quebec farms could move into Ontario. Wild pigs are spreading at a rate of 12.6 km north per year in the United States (Snow et al., 2017) and populations in the Canadian prairies are established (Aschim & Brook, 2019; Brook & van Beest, 2014); wild pigs may eventually invade Ontario from neighboring jurisdictions.

We did not find evidence to support the hypothesis that observed wild pigs are established and self‐sustaining in mainland Ontario. If this were the case, we would have expected to observe larger groups of wild pigs, as seen in places that do have established and growing populations (e.g., Koen et al., 2018; Mayer, 2009; Stolle et al., 2015). Instead, the vast majority of sightings that we have received are of single pigs or very small groups of pigs. Further, conversations with residents and wildlife officials did not reveal any indication of established populations of wild pigs on mainland Ontario or on larger islands such as Manitoulin Island. We acknowledge, however, that just because we have not detected larger groups of wild pigs or wild boar does not mean that they are absent in those places, and therefore, continued vigilance is necessary, especially in areas where we expect that our probability of detection is lower.

We did receive reports indicating that in the past, wild boar have been translocated and illegally released in Ontario to create hunting opportunities. Translocation of wild pigs to create new hunting opportunities is a major factor driving the range expansion of wild pigs in the United States (McCann et al., 2018; Tabak et al., 2017; Waithman et al., 1999). There is increasing evidence from other jurisdictions that removing incentives to hunt wild pigs through prohibitions on transport and hunting, coupled with whole‐sounder removal, is an effective strategy to reduce or eliminate spread (Ditchkoff & Bodenchuk, 2020; Jackling et al., 2016).

Other jurisdictions in North America have also reported that escapes from captive populations of wild boar is a contributing source of invasive wild pigs. In New York, for example, there were correlations between the locations of breeding populations of wild pigs and the location of game farms with wild boar (Jackling et al., 2016). Likewise, Michel et al. (2017) noted that a major escape or release of wild boar from a farm in Saskatchewan was the likely source of current populations of free‐living wild boar nearby. Wild boar escaping captivity and establishing in the wild have also been documented in New Hampshire, Tennessee, North Carolina, and Texas, with anecdotal evidence of similar occurrences in other states (Mayer & Brisbin, 2008). In addition, we received many reports of pot‐bellied pigs and in some cases, these animals had spent several seasons outside of captivity, suggesting that these abandoned pets are capable of surviving in the wild in Ontario. Pot‐bellied pigs contribute to the wild pig population in other jurisdictions as well (e.g., Caudell et al., 2013; Delibes‐Mateos & Delibes, 2013).

We expect that our approach to collecting wild pig sightings may bias the number of wild pigs in each category, as domestic pigs may be more accustomed to human presence, making it more likely to observe them during the day and to record photographic evidence. As such, wild boar may be less likely to be reported and more difficult to verify. The implication is that there may be parts of Ontario with populations of wild boar that we have not yet detected, thus vigilance is still necessary.

Some sightings of wild boar were not near wild boar farms (the maximum distance between a wild boar sighting and a premise with wild boar was 88 km). This suggests that (a) we may not have a full inventory of all wild boar farms in Ontario; (b) that these wild boar were purposely translocated and illegally released; or (c) that they were not recent escapes from farms. These hypotheses are not mutually exclusive, and we do not have the data necessary to tease them apart. It is likely that our dataset does not include the location of all past and present premises that have wild boar, which implies that wild boar sightings are likely even closer to captive wild boar premises than we estimate here. We know that the number of wild boar farms in Ontario decreased between 2001 and 2011 (Michel et al., 2017); many of these farms are missing from our dataset. Likewise, we did not have location data for the source of pot‐bellied pigs, therefore, it is likely that we underestimated the strength of the relationship between domestic pig sightings and their proximity to captive pig premises.

Our findings that observed wild pigs in Ontario are likely recent escapes from captivity, and that we have little evidence at this time that wild pigs are established in Ontario, suggest that Ontario is still early in a potential invasion by wild pigs. Research from other jurisdictions in North America indicates that once invasive wild pigs become established in an area, elimination becomes difficult, if not impossible, and control is expensive. The majority of wild pigs in the United States are hybrids of Eurasian wild boar and western heritage breeds of domestic pigs, implying that wild pigs with at least some Eurasian wild boar ancestry have higher invasive potential (Smyser et al., 2020). As such, efforts focused on removal of wild pigs with at least some Eurasian wild boar ancestry have been successful (e.g., Jackling et al., 2016). Jurisdictions that have been successful at eliminating invasive wild pigs, such as New York and Colorado, USA, have amended regulations to prevent future invasion. These policy tools include prohibiting import, transport, release, and possession of live wild boar, which includes prohibiting the translocation and release of wild pigs to new areas (Centner & Shuman, 2015; Smith, 2020). These successful jurisdictions have also prohibited hunting of wild pigs because allowing wild pig hunting creates an incentive for their illegal release in unoccupied areas for the purpose of creating new hunting opportunities (Bevins et al., 2014). Identifying the source of invasive wild pigs in Ontario can thus help guide management efforts to increase the likelihood of preventing future invasions.

## CONFLICT OF INTEREST

None declared.

## AUTHOR CONTRIBUTIONS

**Erin L. Koen:** Conceptualization (lead); Data curation (supporting); Supervision (equal); Writing‐original draft (lead); Writing‐review & editing (equal). **Erica J. Newton:** Conceptualization (supporting); Data curation (lead); Formal analysis (supporting); Supervision (equal); Visualization (lead); Writing‐review & editing (equal). **E. Hance Ellington:** Conceptualization (supporting); Formal analysis (lead); Writing‐review & editing (equal).

## Data Availability

Distance and group size data: Dryad doi: https://doi.org/10.5061/dryad.5dv41ns6j.

## APPENDIX A

**TABLE A1:** Univariate models predicting the location of wild pig (*Sus scrofa*) sightings in Ontario, Canada, observed between July 2009 and March 2021, grouped by “type” of wild pig, compared to 2,000 random locations

Model^a^	*K*	Wild boar [*n* = 20]			Domestic pig [*n* = 108]			Unknown pig [*n* = 135]		
		AICc	ΔAICc	*w_i_*	AICc	ΔAICc	*w_i_*	AICc	ΔAICc	*w_i_*
Distance to premise with wild boar	2	**207.9**	**0.00**	**1.00**				**986.0**	**0.00**	**0.98**
Distance to domestic pig farm	2				**815.9**	**0.00**	**1.00**	994.2	8.17	0.02
Distance to border (NY)	2	225.2	17.25	0.00	851.2	35.33	0.00	999.3	13.30	0.00
Distance to border (MI‐LP)	2	225.6	17.71	0.00	841.9	26.07	0.00	1,008.2	22.21	0.00
Distance to border (QC)	2	227.0	19.10	0.00	835.2	19.34	0.00	1,006.8	20.81	0.00
Null model	1	226.4	18.49	0.00	854.2	38.32	0.00	1,008.7	22.75	0.00

Note

Univariate models within 2ΔAICc are in bold font.

^a^ Our study area in Ontario shares borders with Quebec, Canada (QC), New York, USA (NY), and the lower peninsula of Michigan, USA (MI‐LP). Ontario also shares borders with Manitoba Canada, Minnesota USA, and the upper peninsula of Michigan USA, but these regions were not included in our study area.

**TABLE A2:** Coefficients [*SE*] from univariate models predicting the location of wild pig (*Sus scrofa*) sightings in Ontario, Canada, observed between July 2009 and March 2021, grouped by “type” of wild pig, compared to 2,000 random locations

Variable^a^	Wild boar [*n* = 20]	Domestic pig [*n* = 108]	Unknown pig [*n* = 135]
Distance to premise with wild boar	**−0.068 [0.019]**		**−0.022 [0.005]**
Distance to domestic pig farm		**−0.156 [0.036]**	**−0.057 [0.017]**
Distance to border (MI‐LP)	**0.002 [0.001]**	**−0.002 [0.001]**	**−0.001 [0.001]**
Distance to border (NY)	**−0.005 [0.003]**	**−0.003 [0.001]**	**−0.004 [0.001]**
Distance to border (QC)	**−0.002[0.002]**	**0.003 [0.001]**	**0.001 [0.001]**

Note

Coefficients with variance that does not overlap zero are in bold font. We measured distances in kilometers.

^a^ Our study area in Ontario shares borders with Quebec, Canada (QC), New York, USA (NY), and the lower peninsula of Michigan, USA (MI‐LP). Ontario also shares borders with Manitoba Canada, Minnesota USA, and the upper peninsula of Michigan, but these areas were not included in our study area.
